# Supplementary material for: Factors associated with retention of mother-baby pairs in the elimination of mother-to-child transmission of HIV program in Kaberamaido district: A longitudinal analysis
Source: PLoS One. 2023 Jul 21;18(7):e0288562. doi: 10.1371/journal.pone.0288562 (PMC10361506; doi:10.1371/journal.pone.0288562)
Supplement: S2 File — (DOCX) [file pone.0288562.s002.docx]

**Supplementary tables using logistic regression models.**

**Factors associated with 12-month retention of mother-baby pairs in Kaberamaido district.**

Table 3 shows factors associated with mother-baby pairs retention at 12months in Kaberamaido district using multiple logistics regression analysis. At multivariate level, mothers’ viral load suppression was the only factor significantly associated with 12-month retention of mother-baby pairs with mothers who had suppressed viral load during pregnancy or breastfeeding having higher odds of 12-month retention compared to mothers who had non-suppressed viral load (adjusted Odds Ratio [aOR]=5.33; 95% Confidence Interval [95%CI]: 1.21, 23.52). All other factors such as age, marital status, place of delivery and mothers ART entry point were not significantly associated with 12-month retention at multivariate level.

**Table 3:** **Factors associated with mother-baby pairs retention at 12-months in Kaberamaido district.**

| **Characteristics** | **Retained at 12 months, n/N(%)** | **Crude**  **OR (95%CI)** | **p-Value** | **Adjusted**  **OR (95%CI)** | **p-Value** |
| --- | --- | --- | --- | --- | --- |
| **Age of the mother** | | | | | |
| <20 Years | 12/16(75.0) | 1.00 |  | 1.00 |  |
| 20-24 Years | 60/63(95.2) | **6.67(1.32,33.69)** | **0.022** | 2.99(0.32,28.40) | 0.340 |
| 25-40 Years | 250/262(95.4) | **6.94(1.95,24.76)** | **0.003** | 3.24(0.43,24.52) | 0.256 |
| >40 Years | 27/27(100.0) | 1.00 |  | 1.00 |  |
| **Marital status** | | | | | |
| Single | 127/136(93.4) | 1.00 |  | 1.00 |  |
| Married/cohabiting | 222/232(95.7) | 1.57(0.62,3.97) | 0.338 | 0.78(0.10,6.20) | 0.813 |
| **Health facility of enrolment** | | | | | |
| HCIII | 190/191(99.5) | 1.00 |  | 1.00 |  |
| Hospital | 159/177(89.8) | **0.05(0.01,0.35)** | **0.003** | 0.12(0.01,1.10) | 0.061 |
| **Mothers’ ART entry point** | | | | | |
| Outpatient Department | 180/188(95.7) | 1.00 |  | 1.00 |  |
| eMTCT | 169/180(93.9) | 0.68(0.27,1.74) | 0.424 | 0.46(0.10,2.10) | 0.314 |
| **Mothers’ treatment supporter** | | | | | |
| Husband | 224/233(96.1) | 1.00 |  | 1.00 |  |
| Other relatives | 125/135(92.6) | 0.50(0.20,1.27) | 0.145 | 0.58(0.07,4.57) | 0.604 |
| **Antenatal care attendance with recent pregnancy** | | | | | |
| No | 21/25(84.0) | 1.00 |  | 1.00 |  |
| Yes | 323/343(95.6) | 4.17(1.27,13.66) | 0.019 | 2.59(0.23,29.54) | 0.443 |
| **Place of delivery of current baby** | | | | | |
| Health facility | 303/320(94.7) | 1.00 |  | 1.00 |  |
| Home | 46/46(95.8) | 1.29(0.29,5.77) | 0.739 |  |  |
| **Viral load monitored** | | | | | |
| No | 128/136(94.1) | 1.00 |  |  |  |
| Yes | 221/232(95.3) | 1.26(0.49,3.20) | 0.634 |  |  |
| **Viral load status (N=232)** | | | | | |
| Non-suppressed | 24/29(82.8) | 1.00 |  | 1.00 |  |
| Suppressed | 197/203(97.0) | **6.84(1.94,24.12)** | **0.003** | **5.33(1.21,23.52)** | **0.027** |
| **Mothers’ ART enrolment time** | | | | | |
| Pre-pregnancy | 160/161(99.4) | 1.00 |  |  |  |
| Antenatal | 176/189(93.1) | **0.08(0.01,0.65)** | **0.018** |  |  |
| Post-natal | 13/18(72.2) | **0.02(0.00,0.15)** | **<0.0001** |  |  |
| **Infant ARV prophylaxis** | | | | | |
| NVP≤72hrs | 267/281(95.0) | 1.00 |  |  |  |
| NVP>72hrs/No ARVs | 82/87(94.2) | 0.86(0.30,2.46) | 0.778 |  |  |
| **Infant age category** | | | | | |
| ≤8weeks | 249/260(95.8) | 1.00 |  | 1.00 |  |
| >8weeks | 100/108(92.6) | 0.55(0.22,1.41) | 0.216 | 0.43(0.10,1.86) | 0.258 |

**=Single, never married, separated, widowed or divorced, **=Married or cohabiting, Bold=Significant with p<0.05, Adjusted OR=Adjusted for all other factors*.

**Factors associated with mother-baby pairs’ cascade completion at 18 months in Kaberamaido district.**

Table 4 shows Factors associated with mother-baby pairs cascade completion at 18 months**.** At bivariate level, age 20-24years (p=0.010) and 25-40years (p=0.005), marital status (p=0.007), health facility level of enrolment (p=0.004), mothers’ ART treatment supporter (p=0.034), viral load status (p=0.023) and mothers’ ART enrolment time (p<0.0001) were significantly associated with mother-baby pair cascade completion at 18-months. At multivariate level, the mothers’ point of ART initiation was significantly associated with cascade completion at 18 months, with mothers who were initiated during the post-natal period having lower odds of cascade completion compared to those who initiated ART before pregnancy (aOR=0.07; 95%CI: 0.02, 0.031). All the other factors had no significant association with cascade completion at multivariate level.

**Table 4: Factors associated with mother-baby pairs cascade completion at 18 months in Kaberamaido district**

| Characteristic | Retained at 18 months, n/N(%) | OR (95%CI) | p-Value | aOR (95%CI) | p -Value |
| --- | --- | --- | --- | --- | --- |
| **Age of the mother** | | | | | |
| <20 | 10/16(62.5) | 1.00 |  | 1.00 |  |
| 20-24 | 57/63(90.5) | **5.70(1.53,21.25)** | **0.010** | 3.96(0.97,16.19) | 0.055 |
| 25-40 | 232/262(88.6) | **4.64(1.57,13.68)** | **0.005** | 2.83(0.87,9.20) | 0.083 |
| >40 | 24/27(88.9) | 4.80(1.00,23.07) | 0.050 | 2.28(0.40,13.14) | 0.355 |
| **Marital status** | | | | | |
| Single* | 111/136(81.6) | 1.00 |  | 1.00 |  |
| Married** | 212/232(91.4) | **2.39(1.27,4.49)** | **0.007** | 2.04(0.84,4.99) | 0.117 |
| **Health facility of enrolment** | | | | | |
| HCIII | 177/191(92.7) | 1.00 |  | 1.00 |  |
| Hospital | 146/177(82.5) | **0.38(0.19,0.73)** | **0.004** | 0.62(0.29,1.34) | 0.228 |
| **Mothers’ ART entry Point** | | | | | |
| OPD | 169/188(89.9) | 1.00 |  | 1.00 |  |
| eMTCT | 154/180(85.6) | 0.67(0.35,1.25) | 0.206 | 0.87(0.40,1.91) | 0.734 |
| **Mothers’ treatment supporter** | | | | | |
| Husband | 211/233(90.6) | 1.00 |  | 1.00 |  |
| Other relatives | 112/135(83.0) | **0.51(0.27,0.95)** | **0.034** | 1.07(0.45,2.54) | 0.884 |
| **Antenatal care attendance of recent pregnancy** | | | | | |
| No | 19/25(76.0) | 1.00 |  | 1.00 |  |
| Yes | 304/343(88.6) | 2.46(0.93,6.54) | 0.071 | 0.98(0.29,3.32) | 0.974 |
| **Place of delivery** | | | | | |
| H/Facility | 280/320(87.5) | 1.00 |  |  |  |
| Home | 43/48(89.6) | 1.23(0.46,3.29) | 0.682 |  |  |
| **Viral load monitored** | | | | | |
| No | 117/136(86.0) | 1.00 |  |  |  |
| Yes | 206/232(88.8) | 1.29(0.68,2.42) | 0.436 |  |  |
| **Mothers’ ART start time** | | | | | |
| Pre-Pregnant | 153/161(95.0) | 1.00 |  | 1.00 |  |
| Antenatal | 160/189(84.7) | **0.29(0.13,0.65)** | **0.003** | 0.40(0.15,1.05) | 0.064 |
| Post-natal | 10/18(55.6) | **0.07(0.02,0.21)** | **<0.0001** | **0.07(0.02,0.31)** | **<0.0001** |
| **Infant ARV prophylaxis** | | | | | |
| NVP≤72hrs | 249/281(88.6) | 1.00 |  | 1.00 |  |
| NVP>72hrs/  No ARVs | 74/87(85.1) | 0.73(0.37,1.47) | 0.378 | 1.26(0.54,2.99) | 0.593 |
| **Infant age at 1st PCR** | | | | | |
| ≤8weeks | 233/260(89.6) | 1.00 |  | 1.00 |  |
| >8weeks | 90/108(83.3) | 0.58(0.30,1.10) | 0.097 | 0.76(0.36,1.60) | 0.469 |

**=Single, never married, separated, widowed or divorced, **=Married or cohabiting, Bold=Significant with p<0.05, Adjusted OR=Adjusted for all other factors*
