# Supplementary material for: Factors associated with retention of mother-baby pairs in the elimination of mother-to-child transmission of HIV program in Kaberamaido district: A longitudinal analysis
Source: PLoS One. 2023 Jul 21;18(7):e0288562. doi: 10.1371/journal.pone.0288562 (PMC10361506; doi:10.1371/journal.pone.0288562)
Supplement: Attachment — Submitted filename: Response to reviewers comments.rtf [file pone.0288562.s003.rtf]

March 22, 2023


The Editor 
PLoS ONE


Dear Sir,


Re: RESPONSE TO COMMENTS RAISED ON MS#: PONE-D-22-29453
Please find enclosed our revised manuscript based on the comments from the Academic Editor and the peer-reviewers.

We are glad for the opportunity to revise the paper, which has improved clarity of the main message in the paper. We thank the reviewers for their insightfulness and the guidance from the Academic Editor.


We look forward to our paper being published in your prestigious journal. Please find below our point-by-point response to the comments, highlighted in red.


Regards,


Dr James Odongo
Corresponding Author


POINT-BY-POINT RESPONSE TO THE ACADEMIC EDITOR AND REVIEWERS' COMMENTS


A: RSPONSE TO ACADEMIC EDITOR COMMENTS

1.	Please ensure that your manuscript meets PLOS ONE's style requirements, including those for file naming. 
Response: The manuscript has been edited to meet PLOS ONE's style requirements.

2.	In your Data Availability statement, you have not specified where the minimal data set underlying the results described in your manuscript can be found. 
Response: The data has been made available, marked, and uploaded online as S2 (Supporting File 2).

3.	We note you have included a table to which you do not refer in the text of your manuscript. Please ensure that you refer to Table 2 in your text; if accepted, production will need this reference to link the reader to the Table. 
Response: Table 2 and 3 have been removed and moved to supplementary tables.

Additional Editor Comments:

1.	Is it a cohort study? By definition, a cohort study requires a clear definition of exposure. Here there is no clear exposure. Yes, there is a follow-up and clear outcomes defined. Please fill out the STROBE checklist form (https://www.strobe-statement.org/checklists/) for cohort studies and annexes. By doing so the co-authors will improve the reporting. 
Response: This is a cohort study, and the exposure was babies being exposed to HIV infection from their mothers. We have re-aligned the work to the STROBE guidelines. A completed copy of the STROBE checklist is attached as S3 (Supplementary File 3).

2.	The background needs some reorganization:
- The paragraph at lines 102 to 107 should come after the paragraph at lines 109 to 114. 
Response: This has been re-aligned. 
- why citation 1 is followed by citations 10 and 11 in the first paragraph? 
Response: Citation numbering has been corrected. 

3.	Lines 150 and 151 - the prevalence here is the overall population of adults, is itn´t? Please be specific. Response: This has been clarified; it's the overall prevalence of HIV among adults. 

4.	Data abstraction procedures: please indicate the full list of variables and do not such lists with etc! Response: The full list of variables has been indicated. (see line 233 to 235 & 242 to 250)

5.	It is late already to correct this. The sample size formula is for just determining a proportion (typically in cross-sectional studies). It is not for looking for association analysis is done here.
Response: We have acknowledged the limitations posed by our sample size formula but as the Editor noted, this is difficult to correct now.

6.	Data analysis and Results:
- It is unclear from the description of the methods whether the authors ascertained the exact follow-up times for each child. That way it would be possible to show a Kaplan-Meier plot of retention. 
Response: Figure 1 shows the Kaplan-Meier plot of retention of the mother baby pairs and the total follow up time has also been summarized in the text.( see page 14) 
- A bit unusual. The follow-up time should be the age of the child because it is at 18 months old that we declare definitely negative or not. Right? Why did the authors decide to use some other follow-up time? 
Response: Indeed, the follow up time was measured in reference to the age of the child and censoring was done for all still active at 18months. 

- The retention is estimated from your sample. Please include 95% confidence intervals. Put those as well in the abstract. 
Response: This was included in the retention and lost to follow up estimates. 

- The calendar time is ignored in this analysis. At least have the year of recruitment as one covariate to add to the model (inlcude it as dummy indicators).
Response: The calendar time was not included in the analysis because time-series analysis focuses on the time to event (how long did the participant take to experience an outcome of interest/failure) and there is staggered entry of participant in the study. 

7.	Table 1: infant age at recruitment please add more descriptives (mean, SD, range, quartiles and keep the dichotomized version as you have). 
Response: This has been included in the table description.

8.	Almost a third of children get to eMTCT past the recommend 8 weeks. We wonder if they do not reach 12 months (after the whole essential immunization for example). This could inform where to act to reduce these losses of opportunity. 
Response: This is true, we have included the observation that a third of the babies receive 1^st^ PCR test late and the need for early identification and enrollment of mother-baby pairs in the eMTCT program under conclusions and recommendations. (see line 445 to 446)

9.	Mothers' ART entry point the eMTCT subdivide (pre-natal, delivery, postdelivery?). 
Response: The entry point of OPD and eMTCT was picked as per the register. However, there is another covariate for the point of ART initiation which is sub divided as pre-ANC (pre-pregnancy), ANC, and postnatal (post-delivery) period, which is included in the analysis. Delivery period could not stand alone as extremely few mothers initiated ART during delivery. 

10.	 Lines 290 to 293: the average time spent by mother-baby pairs in eMTCT program. How this was computed? Does this account for the fact that there is right censoring time here? 
Response: The average time spent by mother-baby pairs in the eMTCT program has been adjusted and survival data summarized as the total follow up time and the average time spent in eMTCT program by those who were lost to follow-up. All mother-baby pairs who were active in care were censured at 18 months.

11.	So the authors decided to study the association at 12 months and at 18 months of follow-up. There were only 19 events (people leaving the cohort) up to the 12th month of follow-up, and another 29 between the 12th and 18th months of follow-up. So for the 12 months, we do not see much when adjusted. Therefore, a) put the current tables 2 and 3 in the annexes. Then do one analysis using a discrete logistic survival model. In this model you have dummy indicators for 12 and 18 months and put the other variables as you have done. Do not include the viral load on the adjusted model. 
Response: Discrete logistic survival model was done and results presented and labeled as table 2. (see page15). The initial table 2 and 3 have been pushed to the annexes as advised. 

12.	Table 2: Add a row for total pairs included. What is the definition for Viral load suppression here? Only 232 mothers had a viral load ie adjusting for this variable you throw away 136 observations. I would recommend not to include this variable. Or do one analysis without this variable and move the current adjusted model with just 232 observations to supplementary materials. 
Response: Viral load has been excluded from the final table with adjusted hazard ratios. 

13.	Figure 1: No need for the bars. Just the line is OK and please add confidence intervals. 
Response: This has been adjusted as a Kaplan-Meier curve with confidence interval included.(see page 14) 


B: RESPONSE TO REVIEWERS' COMMENTS

Reviewer #1: 

1.	What does EID mean? EID (in the abstract and introduction) may even be a universal acronym but it should be explained the first time it is used (which is only found at the study design section). 
Response: EID is early infant diagnosis. This explanation has been included in the abstract and introduction section of the manuscript (see line51).

2.	From how many records were selected the 368 records included in the analysis. 
Response: This was from a sample of 457. All the mother-baby pairs with complete records were included in the analysis and only those with incomplete record excluded. (see page 8)

3.	There is a way to know if there are differences between the pairs included in the analysis compared to those not included. Those not included with incomplete data could be the most missing and lost-to-follow up with a certain implication on the study results. 
Response:  We acknowledge the fact that there could be a difference between the pairs included in analysis compared to those not include. This was a major limitation to our study and has been discussed under study limitations (line 419 to 421). 

4.	The major limitation of the study is the inclusion criterion "On the other hand, clients with incomplete/missing information, clients transferred from other health facilities and clients not in the EID/ART register were excluded from the study", which may have led to a about estimation of results. 
Response: We acknowledge this and have included it as a major limitation to the study. (see line 419 to 421)

5.	Improve formatting and capitalization. 
Response: This has been done.
