# Supplementary material for: Factors associated with retention of mother-baby pairs in the elimination of mother-to-child transmission of HIV program in Kaberamaido district: A longitudinal analysis
Source: PLoS One. 2023 Jul 21;18(7):e0288562. doi: 10.1371/journal.pone.0288562 (PMC10361506; doi:10.1371/journal.pone.0288562)
Supplement: Attachment — Submitted filename: Response to reviewers comments .docx [file pone.0288562.s004.docx]

Jun 12, 2023

The Editor

PLoS ONE

Dear Sir,

**Re: RESPONSE TO COMMENTS RAISED ON MS#:** PONE-D-22-29453

Please find enclosed our revised manuscript based on the comments from the Academic Editor and the peer-reviewers.

We are glad for the opportunity to revise the paper, which has improved clarity of the main message in the paper. We thank the reviewers for their insightfulness and the guidance from the Academic Editor.

We look forward to our paper being published in your prestigious journal. Please find below our point-by-point response to the comments, highlighted in red.

Regards,

**Dr James Odongo**

Corresponding Author

**POINT-BY-POINT RESPONSE TO THE ACADEMIC EDITOR AND REVIEWERS’ COMMENTS**

**A: RSPONSE TO ACADEMIC EDITOR COMMENTS**

Additional Editor Comments:

This report is very relevant as it documents retention rates at 12 and 18 months in the eMTCT (elimination mother-to-child transmission) program somewhere in Uganda. Since the last version there were improvements. There are a few shortcomings:

1. Study design. Be careful with the meaning of the word cohort. Indeed, the authors might have a cohort of mother-children pairs who entered in the eMTCT program and were followed up to 12-18 months. But that alone does not mean it is a cohort (the epidemiologic analytical study) study because a comparison group is not described. Remember in epidemiologic terms an exposure means at least 2 levels (exposed or not exposed) ie. at least a control group (no exposure) must be defined. Which is not the case here. The authors stated in the response to the previous round of comments that "babies being exposed to HIV infection from their mothers" is the exposure. Where is the comparator of such a group? In fact, the analysis never produces an association between retention and "being registered in the eMTCT program". Ok?

Because of this, I suggest changing the title and removing any designation of cohort study here. You may say something like "longitudinal analysis" or "cohort of mother-babies" etc…

Response. The title has been changed and longitudinal analysis adopted.

2. It is still a bit unclear what is the definition to enter the cohort here. Line 184 says "HIV-positive mother-baby pairs enrolled in the eMTCT program from 1st January 2013 to 31st December 2018". When is this event? At PCR sample collection?
Response. The time of entry into the eMTCT program is at the time of 1^st^ PCR sample collection, which is expected to be at 6 weeks from the time of birth. This is the point when the mother-baby pair is recorded into the EID register for follow up. However, the follow up time is always adjusted to consider from the time the infant is born upto the time of exit. Most of the key points in the eMTCT cascade are based on the infant age (eg 1^st^ PCR at 6weeks, 2^nd^ PCR at 12monthsand rapid test at 18months).

3. About the association analysis:
- Please note Cox in "Cox proportional hazard regression" is a name. Please write with capital "C".
- Usually there are 3 time scales to deal in a cohort analysis. The age (child and mother ages), the follow up time and calendar time. The authors i) dealt with the ages (mother age is used for adjustment; child age is homogeneous so OK to ignore for now); ii) the follow up time [which the Cox PH uses, so OK]; iii) Calendar time should not be ignored. For example, the test and treat introduction in 2016 might have changed how and/or characteristics of mother-baby pairs going to eMTCT, and also could affect the retention. Therefore calendar time could be a confounder here. It would be OK to just add dummy indicator of the year the mother-baby entered in the eMTCT. Also add in the table 1 the year of when the pair entered in the eMTCT.

Response. The year of entry into the eMTCT program was included in table1. In table2, the years were merged into “before test and treat” (2013-2016) and “after test and treat”(2017-2018) as test and treat policy was adopted in Uganda in 2016 and implementation started in 2017. This was to ensure that the variable is suitably fitting the regression model (eg in year 2017 alone, there was a cell with a value 1 and this was not suitable to stand alone in the model).

4. Do not only "excel" in line 255. It is "Microsoft excel" and please add a citation and version.

Response. The word Microsoft was added to excel.

5. Line 247 mentions viral load. Of whom? Of the mother or of the child?

Response. This was viral load of the mother and was considered as the latest viral load done during stay in the eMTCT program, for example, a mother who had 2 viral load values, the latest/most current while still in eMTCT program was considered.

6. Figure 2.
- I apologize to the authors that I made them change the time scale to age. Please return to follow up time (in months).
- Add please add below the plot the risk set size.

Response. It is ok to maintain the current scale of age of the infants as the follow up time is based on the age of the infants. As indicated earlier, the time of entry into the eMTCT program is at the time of 1^st^ PCR sample collection, which is expected to be at 6 weeks from the time of birth. This is the point when the mother-baby pair is recorded into the EID register for follow up. However, the follow up time is always adjusted to consider from the time the infant is born upto the time of exit. The duration in eMTCT/follow up time presented here was based on the age of the infant.

7. Figure 1:
- The totals do not add up. This is because we need a box showing the 368 who entered the analysis. So between the 457 and the 368 there were 89 removed.

Response. This has been adjusted accordingly.

8. The 89 who where excluded due to incomplete data represent 19.5% of all potential mother-baby pairs. Please, add a comparison of the 89 and 457.

Response. This has been added as in figure1.


B. RESPONSE TO REVIEWERS' COMMENTS:

**Comments to the Author**

1. If the authors have adequately addressed your comments raised in a previous round of review and you feel that this manuscript is now acceptable for publication, you may indicate that here to bypass the “Comments to the Author” section, enter your conflict of interest statement in the “Confidential to Editor” section, and submit your "Accept" recommendation.

Reviewer #2: (No Response)

2. Is the manuscript technically sound, and do the data support the conclusions?

The manuscript must describe a technically sound piece of scientific research with data that supports the conclusions. Experiments must have been conducted rigorously, with appropriate controls, replication, and sample sizes. The conclusions must be drawn appropriately based on the data presented.

Reviewer #2: Partly

3. Has the statistical analysis been performed appropriately and rigorously?

Reviewer #2: Yes

4. Have the authors made all data underlying the findings in their manuscript fully available?

The [PLOS Data policy](http://www.plosone.org/static/policies.action#sharing) requires authors to make all data underlying the findings described in their manuscript fully available without restriction, with rare exception (please refer to the Data Availability Statement in the manuscript PDF file). The data should be provided as part of the manuscript or its supporting information, or deposited to a public repository. For example, in addition to summary statistics, the data points behind means, medians and variance measures should be available. If there are restrictions on publicly sharing data—e.g. participant privacy or use of data from a third party—those must be specified.

Reviewer #2: Yes

5. Is the manuscript presented in an intelligible fashion and written in standard English?

PLOS ONE does not copyedit accepted manuscripts, so the language in submitted articles must be clear, correct, and unambiguous. Any typographical or grammatical errors should be corrected at revision, so please note any specific errors here.

Reviewer #2: Yes

6. Review Comments to the Author

Please use the space provided to explain your answers to the questions above. You may also include additional comments for the author, including concerns about dual publication, research ethics, or publication ethics. (Please upload your review as an attachment if it exceeds 20,000 characters)

Reviewer #2: The manuscript reports on the very important topic of elimination of mother to child transmission of HIV in Uganda. It focuses on retention of mother-baby pairs, which is critical for the success of any EMTCT program. The manuscript is generally well written; however, the authors may wish to address the following:

Abstract: Line 41-42. The authors may wish to specify that the statistics reported in this sentence are for Uganda. Response. This was included in line 42.

Introduction: Lines 106-107. please add a reference to the transmission risk of 20-25% referred to in this sentence. Response. A reference has been added.

Material and Methods>Study area. Line 153. The authors indicate here that the study was carried out in 5 health facilities in Kaberamaido district. In line 157-158, they indicate that 8 health facilities are providing HIV/AIDS services in the district; and in lines 184-185, the authors state that all HIV-positive mother-baby pairs in the district were enrolled. Is the data presented in the manuscript from 5 or 8 health facilities? Can the authors clarify this in the METHODS section?
The authors should try to provide additional information about the study health facilities. For example, how many of these facilities were health centers and how many were hospitals? What routine PMTCT services are offered in these facilities. When is the first PCR? when is the second PCR? Response. This has been clarified. Data presented here are from 5 health facilities who were accredited to offer ART in the district. The details of the health facilities included have been provided. The routine PMTCT services offered in the facilities have been highlighted in the study area section.

Line 154-169: The authors have provided valuable information about Kaberamaido district. Please cite the source of this information. Response. This information has been cited

Material and Methods>Study Design: Line 180. The follow-up time for the cohort is 24 months. At what point does this follow-up start? is it during pregnancy? At delivery? or some other time? Please clarify. Response. The follow up time has been corrected to 18months. The period is considered to start from the time of birth/delivery of the baby to 18months of age.

The RESULTS indicate that follow-up ends when the baby is 18 months of age; how do the authors reconcile the 18 months with the 24 months stated here? Response. The follow up for the purpose of this study is upto 18months and this has been corrected in the entire document.

Materials and Methods>Sample size determination. The formula used is appropriate for a prevalence study, not this kind of study. This should specifically be mentioned in the limitations. Response. The formula used to calculate the sample size has been acknowledged as a limitation for this study. However, the eventual inclusion into the study was not based on the formula as a census of all those eligible mother-baby pairs in the selected facilities were included in the study.

Materials and Methods>Data abstraction procedures. Line 221-222. The sentence here appears incomplete. Edit it. Response. This has been edited.

Measurement of variables. Line 247-248; Define viral suppression. Response. The viral load suppression was defined as viral load less than 1000 copies/µl of blood.

Line 249-251. Here, the time of Nevirapine initiation is categorized as before or after 24 hours, but in the RESULTS section, Table 1, Nevirapine prophylaxis is categorized as before or after 72 hours. Be consistent in your definition and categorization of this variable. Response. Apologies. Nevirapine initiation was considered as before 72hours or after of birth. This has been corrected to 72hours though out.

DISCUSSION: Line 355-357. TASO-Soroti is mentioned here for the first time without defining it! What does TASO do? What support do they provide to the health facilities? The authors should describe TASO and the kind of work they do in the METHODS section. It is hard to follow the argument in the DISCUSSION without clear understanding of the work done by this organization.
Line 369-372; Health system strengthening by Implementing Partners is given as a possible explanation for the high retention. A brief description of these health system strengthening activities should be written in the METHODS section to give the reader a better understanding of the study setting and routines. To mention this late in the DISCUSSION makes it difficult for the reader to follow the explanations given. Response. A description of TASO, what it does and the health system strengthening project is well documented in the methods section.

Line 381: LTFU is mentioned here and in Table 2. Please write it in full at first mention. Response. The first time LTFU(Lost to follow up) used is in the abstract section and was written in full. Also below the table, it has been written in full.

Line 424-429: The statements here seem to suggest that the authors attempted to reduce misclassification of loss to follow-up by calling participants to determine true outcomes. Participants who could not be traced by phone were left out of the study because of resource limitations (line 428-429). This raises a number of questions. The study was designed to use secondary data from routine facility records, how is it that you added primary data collected through phone interviews? If the phone interviews were carried out for the purpose of collecting research data, did the participants provide any consent? Why did the authors leave out participants who could not be traced? Shouldn't they have been classified as lost to follow-up?

Response. Even though attempts were made by the data collectors to reach out to the mother-baby pairs who were lost, most of them could not be traced and as such the research was restricted to secondary data. We realized that it was also not possible to obtain informed consent from the clients whom we attempted to reach through phone calls and so we did not include the information a few of them provided in this manuscript. The section of attempting to reach out lost participants through phone calls has thus been removed from the text.

Line 437-439: The message in this sentence is not clear. The sentence is probably incomplete. Response. This has been completed.

Table 3>Viral load status. This appears to be the only significant variable in the adjusted model. It would be important to know at what point these viral load results were obtained. Are these results of tests carried out during pregnancy, post-natal period or at any time during study follow-up? How did you define suppression in instances where some participants have 2 or more viral load results in the course of study follow-up? If a test carried out in pregnancy showed viral suppression and later in the post-natal period a subsequent test showed non-suppression or vice-versa, how were these results categorized? Can you clearly define these categories in the METHODS section.

Response. This was viral load of the mother and was considered as the latest viral load done during stay in the eMTCT program, for example, a mother who had 2 viral load values, the latest/most current while still in eMTCT program was considered.
